# Resting-State Brain Anomalies in Type 2 Diabetes: A Meta-Analysis

**DOI:** 10.3389/fnagi.2017.00014

**Published:** 2017-01-31

**Authors:** Wenqing Xia, Yu-Chen Chen, Jianhua Ma

**Affiliations:** ^1^Department of Endocrinology, Nanjing First Hospital, Nanjing Medical UniversityNanjing, China; ^2^Department of Radiology, Nanjing First Hospital, Nanjing Medical UniversityNanjing, China

**Keywords:** type 2 diabetes, resting-state fMRI, meta-analysis, activation likelihood estimation

## Abstract

Resting-state functional magnetic resonance imaging (fMRI) studies have revealed abnormal neural activity in patients with type 2 diabetes mellitus (T2DM). Nonetheless, these findings are heterogeneous and have not been quantitatively reviewed. Thus, we aimed to conduct a meta-analysis that identified consistent results of existing resting-state fMRI studies to determine concordant resting-state neural brain activity alterations in T2DM patients. A systematic search was conducted for resting-state fMRI studies comparing T2DM patients with healthy controls. Coordinates were extracted from clusters with significant differences. The meta-analysis was performed using the activation likelihood estimation method, and nine studies were included. This meta-analysis identified robustly reduced resting-state brain activity in the whole brain of T2DM patients, including the bilateral lingual gyrus, left postcentral gyrus, right inferior temporal gyrus, right cerebellar culmen, right insula and right posterior cingulate cortex (PCC). The present study demonstrates a characteristic pattern of resting-state brain anomalies that will contribute to the understanding of neuropathophysiological mechanisms underlying T2DM.

## Introduction

Type 2 diabetes mellitus (T2DM) is a common metabolic disorder that currently affects approximately 415 million people worldwide, and the prevalence is increasing (International Diabetes Federation, 2015). Patients with T2DM have an increased risk of dementia (Koekkoek et al., 2015), but the neuropathophysiological mechanisms underlying T2DM-related cognitive impairment are not entirely clear. In addition to studies that only rely on neuropsychological performance and experiments based on molecular biology, neuroimaging techniques provide important clues about brain structure, function and metabolism for documenting neurological involvement in T2DM.

Because resting-state functional magnetic resonance imaging (fMRI) is noninvasive and task-free, it has been widely used to investigate the pathogenesis of various neuropsychiatric disorders. Previous resting-state fMRI studies have identified the subtle brain changes located in widespread cortical and subcortical regions that are observed in T2DM patients, but these studies have reported relatively inconsistent results. For instance, several researchers observed reduced neural activity in the cerebellum of T2DM patients (Wang et al., 2014; Peng et al., 2016). In contrast, another study found increased activity in this region (Xia et al., 2013). Furthermore, only one study showed diminished activity in the precentral gyrus (Zhou et al., 2014). These reported discrepancies can potentially be attributed to the limited sample size, variable clinical demographics and use of different methods across studies. Thus, an urgent need exists for a meta-analysis to provide concordant results concerning the role of resting-state anomalies as common markers in T2DM patients.

As a coordinate-based meta-analytic method for meta-analyses of neuroimaging literature, activation likelihood estimation (ALE) can identify brain locations showing a consistent response across multiple experiments. This approach is based on the collection of peak coordinates from each study included in the meta-analysis rather than the input of raw images (Turkeltaub et al., 2002; Laird et al., 2005; Eickhoff et al., 2009). To date, the ALE technique has been successfully applied to neuroimaging studies of various neurological and psychiatric disorders (Shao et al., 2015; Weng et al., 2015). Hence, our present study aimed to quantitatively conduct a meta-analysis using the ALE algorithm to determine the resting-state brain anomalies underlying T2DM.

## Materials and Methods

### Search Strategies and Study Selection

This meta-analysis was performed according to the Meta-analysis of Observational Studies in Epidemiology (MOOSE) criteria (Stroup et al., 2000). A comprehensive literature search up to May 2016 was conducted in PubMed, Web of Knowledge and Embase using the following search terms: (1) “neuroimaging” <OR > “fMRI,” (2) “resting state”; and (3) “type 2 diabetes mellitus” <OR > “type 2 diabetes” <OR > “T2DM”. Our search was restricted to humans. In addition, we reviewed the references cited in articles that were retrieved.

Studies were selected according to the following inclusion criteria: (1) published as an article (not a letter or an abstract); (2) comparisons of T2DM patients with healthy control groups; and (3) clearly reported Montreal Neurological Institute (MNI) or Talairach coordinates of the activation areas (x, y, z). Studies reporting only findings for specific ROIs were not included in the present meta-analysis. For studies that contained two independent patient samples or used two independent methodologies, the appropriate coordinates were extracted as two separate experiments (Cui et al., 2014; Peng et al., 2016). We did not intentionally exclude studies that used a modality other than fMRI or focused on a particular analytic approach. Thus, the methods used in our included studies covered the following approaches: amplitude of low-frequency fluctuations (ALFF), regional homogeneity (ReHo), independent component analysis (ICA), arterial spin labeling (ASL) and degree centrality (DC). Overall, nine fMRI studies (11 experiments) were included in the ALE meta-analysis (Table 1). One study used the ASL perfusion MRI technique to measure resting-state abnormalities in cerebral blood flow in T2DM patients. Two independent reviewers (XW and CYC) evaluated the methodology and the risk of bias of the eligible studies. Any disagreements were assessed by the third reviewer (MJ). The majority opinion was used for the final analysis. We extracted demographic data from each article, including the first author’s name, year of publication, total patient number, sex distribution, patient mean age and range and statistical thresholds.

**Table 1:** **List of all studies included in the meta-analysis: demographic and clinical characteristics of subjects**.

Study	Methodology of analysis	Sample/male		Mean age ± SD		Foci No.	Scanner	SPM	Smoothing kernel (mm)	Statistical threshold	Measure	Tal or MNI
		Patients	Control	Patients	Control
Xia et al. (2013)	ALFF	28/15	29/13	58.7 ± 8.1	57.7 ± 7.2	7	3.0T Siemens	8	4	*P* < 0.05, AlphaSim corrected	GMV	MNI
Cui et al. (2014)	ALFF	29/14	11/27	58.3 ± 7.3	57.8 ± 5.9	7	3.0T Siemens	8	4	*P* < 0.01, AlphaSim corrected	GMV	MNI
Cui et al. (2014)	ReHo	29/14	11/27	58.3 ± 7.3	57.8 ± 5.9	8	3.0T Siemens	8	4	*P* < 0.01, AlphaSim corrected	GMV	MNI
Wang et al. (2014)	ALFF	26/17	26/17	54.7 ± 10.4	54.9 ± 9.8	17	3.0T Phillips Achieva	8	4	*P* < 0.05, AlphaSim corrected	GMV	MNI
Zhou et al. (2014)	ALFF	14/6	17/10	60.50 ± 6.91	63.82 ± 5.79	4	3.0T Siemens	8	8	*P* < 0.05, AlphaSim corrected	GMV	MNI
Cui et al. (2015)	ICA	42/23	42/14	60.4 ± 7.0	58.2 ± 7.3	4	3.0T Siemens	8	4	*P* < 0.05, AlphaSim corrected	GMV	MNI
Xia et al. (2015a)	ASL	38/17	40/21	56.0 ± 6.1	57.1 ± 7.6	5	3.0T Siemens	8	6	*P* < 0.01, FWE corrected	GMV	MNI
Xia et al. (2015b)	ICA	38/21	32/17	58.6 ± 8.2	55.6 ± 7.1	6	3.0T Siemens	8	4	*P* < 0.05, AlphaSim corrected	GMV	MNI
Peng et al. (2016)	ReHo (patients with microangiopathy)	26/12	28/12	57.6 ± 9.3	56.2 ± 6.9	6	3.0T GE Signa Hdxt	8	4	*P* < 0.05, AlphaSim corrected	GMV	MNI
	ReHo (patients without microangiopathy)	22/10	28/12	58.8 ± 7.9	56.2 ± 6.9	6	3.0T GE Signa Hdxt	8	4	*P* < 0.05, AlphaSim corrected	GMV	MNI
Cui et al. (2016)	DC	40/24	43/16	60.5 ± 6.9	57.6 ± 6.6	3	3.0T Siemens	8	4	*P* < 0.05, FWE corrected	GMV	MNI

*Abbreviations: SD, standard deviation; SPM, statistical parametric mapping; MNI, Montreal Neurological Institute; FWE, family-wise error. ALFF, amplitude of low-frequency fluctuations; ReHo, regional homogeneity; ICA, independent component analysis; ASL, arterial spin labeling; DC, Degree centrality*.

### Data Extraction and Coordinate-Based Meta-Analysis

The x, y and z peak activation coordinates of all eligible contrasts constituted the meta-analysis input. The MNI coordinates were recorded and implemented in GingerALE 2.3.3^1^ (Research Imaging Institute of the University of Texas Health Science Center, San Antonio, TX, USA).

Ginger ALE software was used to compare the brain changes between T2DM patients and healthy controls. The reported loci of maximal anatomical differences were modeled as the peaks of three-dimensional Gaussian probability density functions defined by the full-width at half-maximum (FWHM), which was set according to a quantitative uncertainty model (Laird et al., 2005; Eickhoff et al., 2009). ALE values were calculated on a voxel-by-voxel basis by measuring the union model activation (MA) maps modeled above. This revised analysis tested for convergence by study (random effects) instead of foci (fixed effects). These maps were finally thresholded at *P* < 0.05 and corrected for multiple comparisons using the false-discovery rate (FDR; q; Genovese et al., 2002). The volume of the minimum cluster threshold was set at 200 mm^3^. The coordinates of the weighted center were generated for each cluster. The resulting significant anatomical areas were labeled based on probabilistic cytoarchitectonic maps of the human brain using the SPM Anatomy Toolbox v2.1 (Eickhoff et al., 2005). The results were visualized with Mango software^2^ using the Colin brain template in the MNI space^3^.

## Results

**Figure 1:**
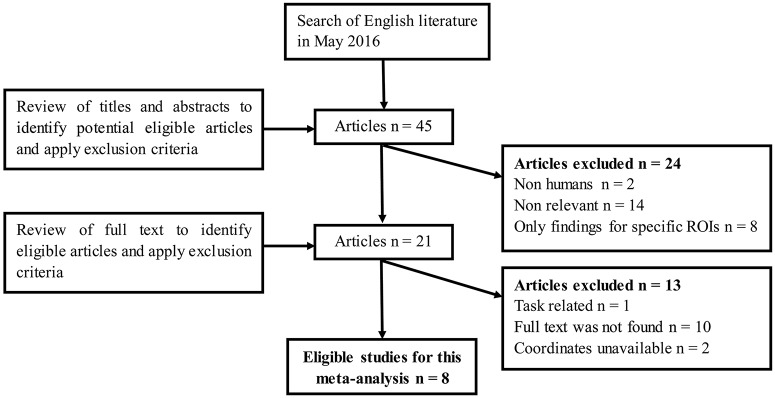
**Flow diagram of the literature search.** The flow diagram shows the results of the systematic search for the selected studies in this meta-analysis.

We identified nine eligible studies (Xia et al., 2013, 2015a,b; Cui et al., 2014, 2015, 2016; Wang et al., 2014; Zhou et al., 2014; Peng et al., 2016) for the meta-analysis according to the search criteria mentioned above. In one study, the analysis was performed based on two different subgroups of T2DM patients who were then compared with the same healthy control group (Peng et al., 2016). In another study, the analysis was performed with two different methodologies (Wang et al., 2014). Therefore, we treated these studies as unique reports, with each patient subgroup included independently in the meta-analysis; therefore, a total of 11 datasets were ultimately included in the meta-analysis. Figure 1 shows the details of the literature search strategy and data extraction process. The clinical and demographic data of the participants from all recruited studies are presented in Table 1. The patients and controls from each study were generally matched by age, gender and education.

**Figure 2:**
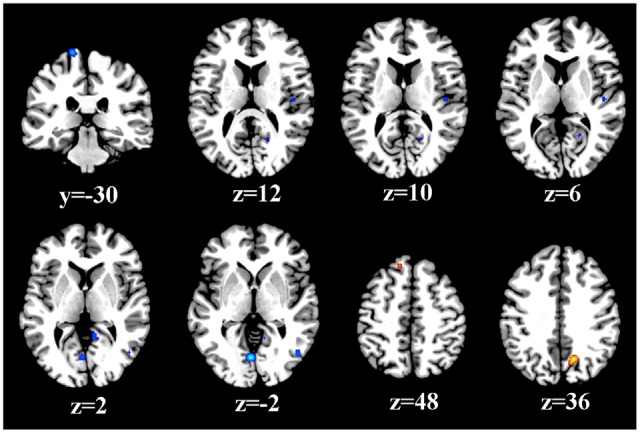
**Resting-state brain activity alterations in T2DM patients compared to healthy controls.** The results are from the ALE meta-analyses are shown. All activations are significant at *P* < 0.05 and corrected for multiple comparisons using FDR correction.

As illustrated in Figure 2, a total of 73 peak foci were reported in this meta-analysis. Compared to healthy controls, T2DM patients had widespread reduced resting-sate neural activity in the whole brain, including the bilateral lingual gyrus, left postcentral gyrus, right inferior temporal gyrus, right cerebellar culmen, right insula and right posterior cingulate cortex (PCC). However, enhancements were also observed in the right precuneus and left superior frontal gyrus. Table 2 displays the coordinates of the cluster maxima.

**Table 2:** **Resting-state anomalies in T2DM patients compared with healthy controls**.

Brain regions	BA	MNI coordinates *x, y, z* (mm)	ALE extrema value	Cluster size (mm^3^)
**(1) Decrease in T2DM**
L Lingual Gyrus	18	−4, −74, −2	0.0188	832
L Postcentral Gyrus	3	−16, −30, 76	0.0154	432
R Inferior Temporal Gyrus	37	48, −70, −2	0.0126	328
R Cerebellar Culmen	–	10, −50, 2	0.0118	328
R Insula	13	46, −18, 10	0.0105	272
R Lingual Gyrus	18	20, −58, 6	0.0097	216
R Posterior Cingulate Cortex	30	18, −62, 12	0.0096	
**(2) Increase in T2DM**
R Precuneus	7	16, −64, 36	0.0138	656
L Superior Frontal Gyrus	8	−12, 38, 48	0.0107	320

*Abbreviations: BA, Brodmann area; MNI, Montreal Neurological Institute; ALE, activation likelihood estimation; L, left; R, right*.

## Discussion

The current study is the first meta-analysis to explore resting-state brain abnormalities in T2DM patients. By analyzing nine eligible studies, this meta-analysis identified consistent regions of resting-state activity brain anomalies across almost the entire brain of T2DM patients, including the frontal, parietal, sensorimotor, temporal, occipital and insular cortices. Therefore, T2DM-related neuropsychiatric impairment is believed to be correlated with diffuse aberrant resting-state brain activity involving both cortical and subcortical structures, and these findings will allow for a more comprehensive understanding of neuropathological mechanisms of this disorder.

Consistent with previous brain perfusion studies without resultant coordinates (Novak et al., 2006), parietal and occipital regions were found to be significantly altered in this meta-analysis. As a key region of the visual cortex, the occipital gyrus is the dominant region that exhibited remarkable reduction of resting-state neural activity in T2DM patients, consistent with the idea that visuospatial dysfunction is a common manifestation in T2DM patients (Moran et al., 2013). Worse visuospatial performance has been observed in T2DM patients than in their matched controls (Bangen et al., 2015), which could be due to the deficits in this area. Furthermore, the postcentral gyrus is known to be the main sensory receptive region for the sense of touch, proprioception, pain and temperature, which could contribute to common diabetic limb pain and temperature sensation decline (Cauda et al., 2009). In fact, impaired sensorimotor function has been described in T2DM patients with peripheral neuropathy (Menz et al., 2004). Nevertheless, the cause-and-effect relationship between deactivation in the postcentral gyrus and diabetic neuropathy cannot be explained at this stage because all the included studies were cross-sectional.

The temporal lobe has been linked to memory, verbal fluency, language processing and speech production, which are impaired in T2DM patients (McCrimmon et al., 2012). T2DM patients showed an abnormal pattern in the inferior temporal gyrus in the current study, which is also consistent with a previous voxel-based morphometry (VBM) study that revealed that T2DM patients exhibited gray matter atrophy in the right temporal lobe (Chen et al., 2012). Although it has been assumed that functional activity changes could precede or lead to subtle brain structural abnormalities, the relationship between functional and structural alterations should be further clarified by longitudinal studies. Several hypothesis-driven MRI studies have also demonstrated the vulnerability of the temporal lobe in T2DM patients. By seeding the PCC during a resting-state fMRI scan, two different research groups reported decreased functional connectivity within the temporal lobe (Musen et al., 2012; Chen et al., 2014). Similarly, by seeding the bilateral hippocampus, abnormal connections within the temporal lobe have also been found. A subtle effect of T2DM on cortical atrophy has also been observed in this area (Brundel et al., 2010). These aforementioned findings provide evidence of disrupted temporal functioning in T2DM patients and suggest that temporal dysfunction should be evaluated when cognitive decline occurs in T2DM patients.

Reduced resting-state brain activity in the cerebellum and insula may also contribute to cognitive deficits in T2DM patients. The cerebellum controls not only motor coordination but also advanced cognitive function (Marklund et al., 2007). Given that the salience network has been found to be impaired in T2DM patients (Chen et al., 2015), and the insula acts as an integral area in the salience network (Menon and Uddin, 2010), decreased insula activity could lead to impaired generation of appropriate behavioral responses to salient stimuli in these patients. Interestingly, according to the results, the right hemisphere of T2DM patients is more seriously impaired than the left hemisphere. Likewise, the effects of T2DM on the cortical surface and volume were only significantly pronounced in the right hemisphere (Brundel et al., 2010). The right hemisphere has been determined to be dominant over the left in geometric analysis and visual-spatial perceptual functioning, including the perception of distance, direction, shape, position, orientation and the detection of complex and hidden figures (Joseph, 1988); therefore, visual-spatial perceptual disturbances, which have been described in T2DM-related cognitive decline, could arise due to right hemisphere impairment. Coincidentally, according to some results extracted from the studies included in this meta-analysis, performance on one neuropsychological test that reflects visual-spatial function, the Rey-Osterrieth Complex Figure Test, has been observed to be correlated with the neural activity in the right brain region (Xia et al., 2015a). Nonetheless, because most studies in this field observed brain alterations on both sides in T2DM patients, the lateralization of impairment of brain activity in T2DM patients requires further investigation.

An additional finding of the present meta-analysis was hyperactivity in the frontoparietal cortex. The precuneus is involved in visuospatial function and working memory, and the frontal lobe is considered as a critical region involved in executive function and attention that subserves cognitive and emotional processing (Radua et al., 2012). These enhanced activities could act in a compensatory or recruitment capacity in T2DM patients such that their cognitive performance could be retained to some extent.

Several limitations should be noted in this meta-analysis. First, the number of included studies was relatively small. Second, ALE can provide excellent control of false positive results, but false negatives are more difficult to avoid (Radua et al., 2012). Finally, heterogeneity of the included studies, such as the demographics of the patients and different imaging modalities that represented different aspects of resting-state abnormalities, could not be entirely excluded. For example, ALFF and ReHo are relevant to the intensity and temporal synchronization of regional spontaneous neuronal activity in the whole brain, respectively (Biswal et al., 1995; Zang et al., 2004). ICA decomposes the entire resting-state BOLD data set into spatially distributed components and automatically identifies meaningful brain networks (Mantini et al., 2007), and ASL perfusion MRI measures regional cerebral blood flow and reflects regional brain metabolism and neural activity (Detre and Alsop, 1999). Despite these differences, various analytic approaches might be complementary to each other and provide more comprehensive information. Similar patterns of resting-state anomalies could also be detected by different analytic modalities. For instance, reduced resting-state activity in the occipital lobe was found in most of our included studies, regardless of the imaging modality.

In summary, this meta-analysis demonstrated robust resting-state brain anomalies in T2DM patients compared to healthy controls that were widespread in the frontal, parietal, sensorimotor, temporal, occipital and insular cortex. Further large, multicenter, resting-state fMRI studies are required to comprehensively investigate whether this abnormal activity pattern is a valuable diagnostic and prognostic biomarker for T2DM-related neuropsychiatric impairment.

## Author Contributions

WX and Y-CC performed the analysis and wrote the manuscript. JM designed the meta-analysis and revised the manuscript.

## Conflict of Interest Statement

The authors declare that the research was conducted in the absence of any commercial or financial relationships that could be construed as a potential conflict of interest.

## Footnotes

^1^http://brainmap.org/ale/

^2^http://ric.uthscsa.edu/mango/mango.html

^3^http://www.brainmap.org/ale
